# PLGA Nanoparticles Uptake in Stem Cells from Human Exfoliated Deciduous Teeth and Oral Keratinocyte Stem Cells

**DOI:** 10.3390/jfb13030109

**Published:** 2022-07-31

**Authors:** Maria Tizu, Ion Mărunțelu, Bogdan Mihai Cristea, Claudiu Nistor, Nikolay Ishkitiev, Zornitsa Mihaylova, Rozaliya Tsikandelova, Marina Miteva, Ana Caruntu, Cristina Sabliov, Bogdan Calenic, Ileana Constantinescu

**Affiliations:** 1 Centre for Immunogenetics and Virology, Fundeni Clinical Institute, Carol Davila University of Medicine and Pharmacy, 258 Fundeni Road, 022328 Bucharest, Romania; maria.tizu@drd.umfcd.ro (M.T.); ion.maruntelu@drd.umfcd.ro (I.M.); ileana.constantinescu@imunogenetica.ro (I.C.); 2 Department of Anatomy, Carol Davila University of Medicine and Pharmacy, 8 Blvd Eroii Sanitari, 050474 Bucharest, Romania; bogdan.cristea@umfcd.ro; 3 Central Military Hospital, Carol Davila University of Medicine and Pharmacy, 134 Stefan Furtuna Street, 010899 Bucharest, Romania; claudiu.nistor@umfcd.ro; 4 Department of Medical Chemistry and Biochemistry, Medical Univerity of Sofia, Blvd Akademik Ivan Evstratiev Geshov 15, 1431 Sofia, Bulgaria; ishkitiev@gmail.com (N.I.); m.miteva@medfac.mu-sofia.bg (M.M.); 5 Department of Oral and Maxillofacial Surgery, Medical Univerity of Sofia, Blvd Akademik Ivan Evstratiev Geshov 15, 1431 Sofia, Bulgaria; dr.z.mihaylova@gmail.com; 6 Biosciences Institute, Newcastle University, Catherine Cookson Building, Newcastle upon Tyne NE2 4HH, UK; mstsikandelova@gmail.com; 7 Department of Oral and Maxillofacial Surgery, “Carol Davila” Central Military Emergency Hospital, 010825 Bucharest, Romania; ana.caruntu@gmail.com; 8 Department of Oral and Maxillofacial Surgery, Faculty of Dental Medicine, “Titu Maiorescu” University, 031593 Bucharest, Romania; 9 Biological and Agricultural Engineering Department, Louisiana State University and LSU Agricultural Center, 141 E. B. Doran Bldg, Baton Rouge, LA 70803, USA

**Keywords:** PLGA nanoparticles, oral keratinocyte stem cells, human exfoliated deciduous teeth

## Abstract

Polymeric nanoparticles have been introduced as a delivery vehicle for active compounds in a broad range of medical applications due to their biocompatibility, stability, controlled release of active compounds, and reduced toxicity. The oral route is the most used approach for delivery of biologics to the body. The homeostasis and function of oral cavity tissues are dependent on the activity of stem cells. The present work focuses, for the first time, on the interaction between two types of polymeric nanoparticles, poly (lactic-co-glycolic acid) or PLGA and PLGA/chitosan, and two stem cell populations, oral keratinocyte stem cells (OKSCs) and stem cells from human exfoliated deciduous teeth (SHEDs). The main results show that statistical significance was observed in OKSCs uptake when compared with normal keratinocytes and transit amplifying cells after 24 h of incubation with 5 and 10 µg/mL PLGA/chitosan. The CD117^+^ SHED subpopulation incorporated more PLGA/chitosan nanoparticles than nonseparated SHED. The uptake for PLGA/chitosan particles was better than for PLGA particles with longer incubation times, yielding better results in both cell types. The present results demonstrate that nanoparticle uptake depends on stem cell type, incubation time, particle concentration, and surface properties.

## 1. Introduction

PLGA or poly (lactic-co-glycolic acid), a copolymer approved by the U.S. Food and Drug Administration, is currently used in a broad range of applications in the medical field as a delivery system for biologics due to its biologic properties such as biocompatibility and biodegradability. Various therapeutic agents have been encapsulated within PLGA nanoparticles (PLGA-NP), as the polymeric matrix preserves the drugs and prevents degradation [1,2]. Depending on the tissue or system, the internalization of PLGA nanoparticles can be optimized by adjusting their size, charge, concentration, and incubation time. 

In this context, oral delivery is a widely used approach for the delivery of biologics to the body, with the oral cavity being the first component of the gastrointestinal tract [3]. However, the impact of PLGA nanoparticles on oral cells, and especially oral stem cell populations, has received little attention. Therefore, their potential to act as a viable and safe delivery mechanism for oral drug administration requires further research. PLGA-NP efficiency as a carrier also depends on the oral microenvironment, which may impact the rate of nanoparticle uptake and disintegration. It is thus important to measure a baseline for which these nanoparticles are internalized by oral stem cell populations under in vitro, tightly controlled, conditions.

It is well-established that maintaining oral mucosal architecture and homeostasis is heavily dependent on the proper function of oral keratinocyte stem cells (OKSCs) [4]. Due to their importance and potential roles in regenerative medicine, several attempts have been made to isolate and characterize OKSCs. However, these cells are difficult to isolate, mainly due to the lack of appropriate surface markers. Previously, our team successfully isolated a pure subpopulation of OKSCs using two surface markers on α6β4 integrin and CD71 and a magnetic method for cell sorting. In our previous studies, we also showed that epithelial cells with the phenotype α6β4posCD71neg have important stem-cell attributes and can be successfully expanded in vitro [5].

Mesenchymal stem cells (MSCs) are already routinely separated from dental tissues. These cells are capable of, in appropriate conditions, differentiating into various cell types: adipocytes, chondrocytes, osteoblast-like cells, etc. Dental MSCs play important roles in tissue homeostasis, as they are able to repair damaged hard and soft oral structures, such as dentine and periodontal ligament [6]. To date, several types of dental-tissue-derived MSCs have been investigated: dental pulp stem cells (DPSCs), stem cells from human exfoliated deciduous teeth (SHEDs), periodontal ligament stem cells (PDLSCs), progenitor cells from dental follicles (SCDFs), and stem cells from apical papilla (SCAPs) [7,8]. Dental-derived stem cells may be the best cell source for transplantation. However, various in vitro and preclinical studies should be performed. It is well-known that the MSCs of stem cell surface markers expression include CD105, CD146, CD90, etc. In our previous studies, we successfully performed magnetic separation of DPSCs and SHEDs using a CD117 stem cell marker. Our results demonstrate that CD117+ cells possess strong potential for pancreatic differentiation in appropriate conditions, and may therefore have applications in future pancreatic disorders therapy [9].

To the best of our knowledge, nanoparticle interaction with the oral environment, particularly with oral mucosa, has been insufficiently explored. Our hypothesis in this study was that nanoparticle cellular uptake depends on several variables, such as stem cell type, length of incubation, concentration of nanoparticles, and particle surface properties. In this context, the specific aim of the present work was to assess the interactions between OKSCs, SHEDs, and PLGA nanoparticles with different attributes and to determine their uptake parameters. 

## 2. Materials and Methods

### 2.1. Materials

PLGA (or poly (lactic-co-glycolic) acid at 40–75 g/mol, used 50:50), chitosan (Chi) (100–300 g/mol), polyvinyl alcohol (PVA) (31–50 g/mol) 87–89% hydrolyzed, fluorescein isothiocyanate (FITC), N-Boc-ethylenediamine, N,N-diisopropylethylamine (DIPEA),trifluoroacetic acid (TFA), 1-[Bis(dimethylamino)methylene]-1H-1,2,3-triazolo [4,5-b]pyridinium 3-oxid hexafluorophosphate) (HATU), 4′,6′-diamidino-2-phenylindole (DAPI), boric acid, and potassium iodine were acquired from Sigma-Aldrich (St. Louis, MO, USA). HPLC-grade ethyl acetate, acetone, DCM, ethanol, and acetonitrile were purchased from Mallinckrodt Baker (Pittsburgh, NJ, USA).

### 2.2. PLGA-FITC Conjugation

The fluorescent nanoparticles were synthesized by covalently linking FITC to PLGA. Briefly, PLGA (2 g) was dissolved in DCM (35 mL) at room temperature, and 48 mg N-Boc-ethylenediamine, 100 mg HATU, and 0.15 mL DIPEA were added under gentle stirring. After 10 h, the reaction was stopped by adding water (100 mL). PLGA-NH-Boc precipitate was obtained. 

### 2.3. Nanoparticle Synthesis

The polymeric nanoparticles of PLGA, PLGA-FITC, and PLGA/Chi were obtained using an emulsion solvent evaporation method [10]. In short, PLGA and PLGA-FITC (400 mg) were dissolved in ethyl acetate. The solution was then added to 2% (*w/v*) 120 mL PVA aqueous solution. Ethyl acetate was further used for saturation of the aqueous phase.

The emulsion was mixed for 60 s and homogenized in a microfluidizer (M110P apparatus, Microfluidics, Westwood, MA, USA) at 4 °C and 30,000 psi for a total of 3 times. Next, the solvent was evaporated using a rotary evaporator (Buchi Inc., New Castle, DE, USA) for 1 h. In order to remove free PVA, the NPs suspension was then dialyzed with 100 kDa cut-off membrane (Spectrum Laboratories, Rancho Dominguez, CA, USA) for 48 h. Every 8 h, distilled water was replaced. The PLGA/Chi polymeric nanoparticle synthesis followed the same protocol previously described, but the sample after dialysis (10 mL) was mixed with 10 mL of 2 mg/mL Chi solution at pH 5 for 10 min. In the last step, nanoparticles were combined 1:1 (*w/w*) with trehalose and lyophilized for 2 days at −80 °C using a Labconco freeze dryer (Kansas City, MO, USA). NPs were stored at −20 °C.

### 2.4. Nanoparticle Characterization

NP characteristics such as size (diameter mean), polydispersity index (PDI), and zeta potential were acquired using dynamic light scattering (DLS; Zetasizer Nano, Malvern Instruments, Southborough, MA, USA). Working conditions were set as follows: sample dilution 1:20 (*v/v*) in HPLC-grade water, pH = 5, and temperature 25 °C.

Nanoparticle morphology was observed using a transmission electron microscope (TEM; JEM-1400 system, JEOL, Peabody, MA, USA). NPs were stained using 2% uranyl acetate; for analysis, the nanoparticle suspension was placed on 400-mesh carbon-coated grids.

A colorimetric assay was used to quantify the PVA remaining after nanoparticle dialysis [11]. In short, NPs powder was resuspended in 2 mL of 0.5 N NaOH and heated for 15 min at 60 °C. The samples were neutralized using 0.9 mL of 1 N HCl, and distilled water was added until a final volume of 5 mL. In the next step, 1.5 mL of distilled water, 0.5 0.05 M/0.15 M I_2_/KI, and 3 mL of 0.65 M boric acid completed a volume of 10 mL. After 15 min of incubation at room temperature, the samples were measured using a spectrophotometer (Fisher Scientific, Pittsburg, PA, USA) at 690 nm. The standard curve for PVA was obtained using the same protocol for a concentration of 2.5–60 µg/mL.

### 2.5. Cell Culture

Isolation and separation of oral keratinocyte stem cells were performed following a protocol developed by our team and described in detail in [12]. Deciduous teeth and mucosa were supplied by patients undergoing routine deciduous tooth extraction at Dental Faculty Hospital, Medical University, Sofia, Bulgaria. All patients participating in the study signed an informed consent form following the decision of the Ethical Committee of Medical University, Sofia’s Council of Medical Science (No. 4770\11.12.2018). Tissues were thoroughly rinsed for blood and debris with PBS, followed by enzymatic dissociation with 4 mg/mL dispase II (Sigma Aldrich, St. Louis, MO, USA) and 3 mg/mL collagenase (Sigma Aldrich, St. Louis, MO, USA) for 2 h at room temperature. Epidermal sheets were then removed from the dermal side, treated with trypsin 0.025% for 30 min, and centrifuged. Primary keratinocytes were cultured in EpiLife culture medium (Cascade Biologics, Portland, OR, USA) together with 1.2 mM of calcium and antibiotics—fungizone and kanamycin. The pulp was accessed through the resorbed tooth root canal and extracted by sterile barbed broach. Pulps were collected in DMEM (Invitrogen, Eugene, OR, USA) supplemented with 10% FBS (Sigma-Aldrich, St. Louis, MO, USA) and 1% antibiotic–antimycotic (Sigma-Aldrich) in sterile containers up to 24 h after extraction at 4 °C. The pulp was then digested in a PBS (Lonza, Verviers, Belgium) solution of 3 mg/mL collagenase type I/4 mg/mL dispase II for 1 h at 37 °C. Pulps were seeded in 2 cm^2^ petri dishes (Greiner Bio-One, Frickenhausen, Germany) containing DMEM supplemented with 1% antibiotic–antimycotic and 20% FBS. Cell cultures from human exfoliated deciduous teeth pulp (SHEDs) were incubated in humidified atmosphere of 5% CO_2_ and 95% air at 37 °C for a period of 2 to 4 weeks. The medium was removed, and a fresh one was added every second day until a monolayer of confluent cells was reached. Cells were observed using a phase contrast microscope (Leica Microsystems GmbH, Germany). After reaching 60–70% confluence, the cells were transferred to larger tissue culture flasks for further culturing. Prior to magnetic separation, the cells were expanded for 3 passages.

### 2.6. Magnetic Separation

Primary keratinocyte cells were further subjected to magnetic separation based on two markers expressed on the cell membrane surface: integrin α6β4 and CD71. In the first step of the separation, the cells were incubated with integrin α6β4 antibody and IgG MicroBeads. The obtained cell cocktail was placed in the magnetic field of a MACS Separator (Biotec Inc., CA, USA). Cells expressing integrin α6β4 were retained in the working column and represented the positive fraction. In the second step, this fraction was reacted with CD71 MicroBeads and subjected to the same procedure as the first isolation. Following magnetic isolation, we obtained three cell subpopulations: α6β4negative postmitotic keratinocyte cells, α6β4positive CD71positive transit amplifying cells, and α6β4 positive CD71negative fraction oral keratinocyte stem cells. Cells were routinely grown at 37 °C in 5% CO_2_ in flasks precoated with collagen IV at 20 µg/mL (Sigma) and passaged at 60% confluence.

SHEDs were further subjected to magnetic separation, as described above, based on CD117 expression. The CD117^+^ cell fraction was routinely grown at 37 °C in 5% CO_2_, passaged at 60% confluence, and seeded in 24-well plates (Sigma).

### 2.7. Immunofluorescence

OKSCs, transit amplifying cells, and postmitotic keratinocyte cells were cultured at a density of 1 × 10^4^ cells/well into four chamber slides (NalgeNuncInt, Naperville, IL, USA). The cells were exposed to different concentrations of PLGA and PLGA/Chi NPs: 1, 5, and 10 µg/mL for various periods of time: 3, 12, and 24 h. Following NPs exposure, each well was washed thoroughly 3–5 times with PBS and fixed in 4% paraformaldehyde, and then reacted with saturated levels of DAPI (blue) used for staining cellular nuclei. For visualization purposes, all NPs were previously conjugated with FITC (green). For semiquantitative analysis, five high-power (20× magnification) fields were randomly chosen for each slide. Cells with clearly defined staining were counted, and the count was divided by the total number of cells in each field.

Nonseparated and CD117^+^ SHEDs seeded at a 1 × 10^4^ cells/cm^2^ density into 24-well plates were exposed to PLGA and PLGA/Chi NPs containing media at the same conditions mentioned above. After the end of the exposure time, the media containing nanoparticles was removed, cells were washed with PBS, and normal growth media without nanoparticles were introduced. The immunofluorescence images of the nanoparticle treated cells were acquired using an IN Cell Analyzer 6000 (GE Healthcare, Buckinghamshire, UK). We photographed 25 fields in each well. The green fluorescence intensity of between 1.5 × 10^3^ and 3 × 10^3^ cells in each well was analyzed using image analysis software (IN Cell Developer Toolbox 1.7, GE Healthcare). Cells showing values greater than 0.065 for total cytoplasmic green fluorescence were counted and compared with the total number of cells analyzed.

### 2.8. Statistical Analysis

The results from twenty-five fields of view for each well are presented as means ± standard deviations. Statistical analysis between different time groups and different concentration groups was performed by using one-way analysis of variance (ANOVA) (SigmaPlot 13; Systat Software GmbH, Erkrath, Germany). Statistical significance was accepted at *p* < 0.05.

## 3. Results

**Figure 1:**
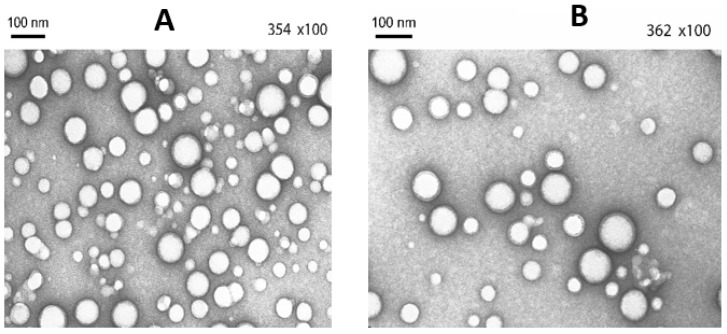
TEM pictures of (**A**) PLGA and (**B**) PLGA/Chi nanoparticles.

PLGA and PLGA/Chi polymeric nanoparticles were spherical in shape (Figure 1) and measured 95 nm (PLGA NPs) to 132 nm (PLGA/Chi NPs) (Table 1). The size distribution was narrow for PLGA NPs (PDI = 0.12), whereas PLGA/Chi NPs showed a wider distribution (PDI = 0.38). PLGA NPs had a negative zeta potential (−38 mV), whereas PLGA/Chi NPs were positive (+76 mV) due to the presence of chitosan on the surface (Table 1). Both particles were covered with a similar amount of PVA (25%).

In the present study, three subpopulations of isolated keratinocytes and two subpopulations of SHED were exposed to 1, 5, and 10 µg/mL of PLGA and PLGA/chitosan NPs for various time intervals: 3, 12, and 24 h.

**Figure 2:**
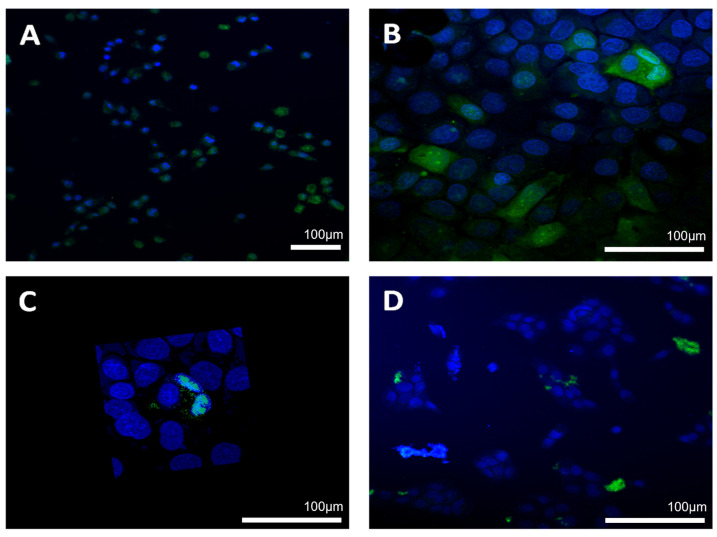
Immunofluorescence: oral keratinocyte stem cells uptake of PLGA/chitosan nanoparticles following 24 h exposure (blue DAPI staining was used for nuclei, while NPs are labelled with FITC and appear in green): (**A**) ×20 magnification; (**B**) ×40 magnification; (**C**) 3D model; (**D**) NPs outside cell membrane; nanoparticle concentration—5 µg/mL.

**Figure 3:**
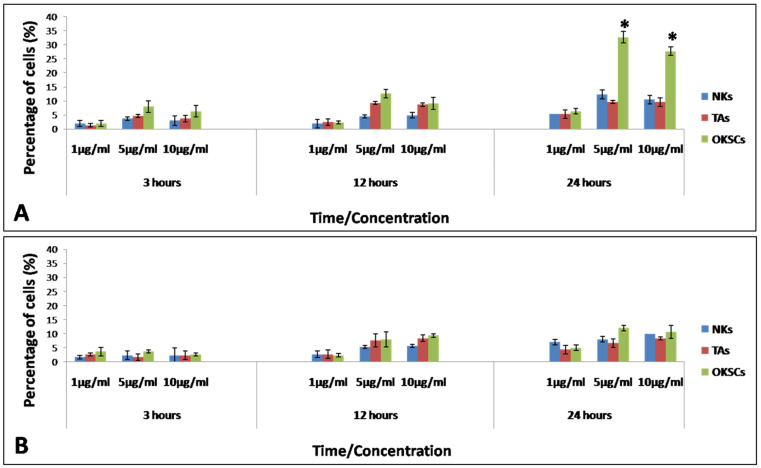
Uptake of polymeric nanoparticles by different oral keratinocyte cells at various NPs concentrations and different time points. (**A**) PLGA/chitosan uptake by normal keratinocytes (NKs), transit amplifying cells (TA), and oral keratinocyte stem cells (OKSCs); (**B**) PLGA uptake by normal keratinocytes (NKs), transit amplifying cells (TAs), and oral keratinocyte stem cells (OKSCs). Statistical significance was set at * *p <* 0.05 ANOVA; n = 5 independent experiments.

The percentage of cells containing fluorescent nanoparticles increased with exposure time and concentration of nanoparticles, but the increase was not significant, with one exception (Figure 2). A significant statistical difference was observed for OKSCs compared with NKs and TA cells, when incubated with 5 and 10 µg/mL PLGA/chitosan after one day of incubation (Figure 3A): for 5 µg/mL: 32.67 ± 2.08 vs. 9.67 ± 0.58 vs. 12.33 ± 1.53 for OKSCs, TA, and NKs, respectively; and for 10 µg/mL: 27.67 ± 1.53 vs. 9.63 ± 1.53 vs. 10.52 vs. 1.53 for OKSCs, TA, and NKs, respectively (data presented as means ± SD; *p* < 0.05. ANOVA, n = 5 independent experiments). Similarly, no statistical increases were detected in keratinocyte cells incubated with PLGA NPs at concentrations of 1, 5, and 10 µg/mL for 3, 12, and 24 h (Figure 3B).

**Figure 4:**
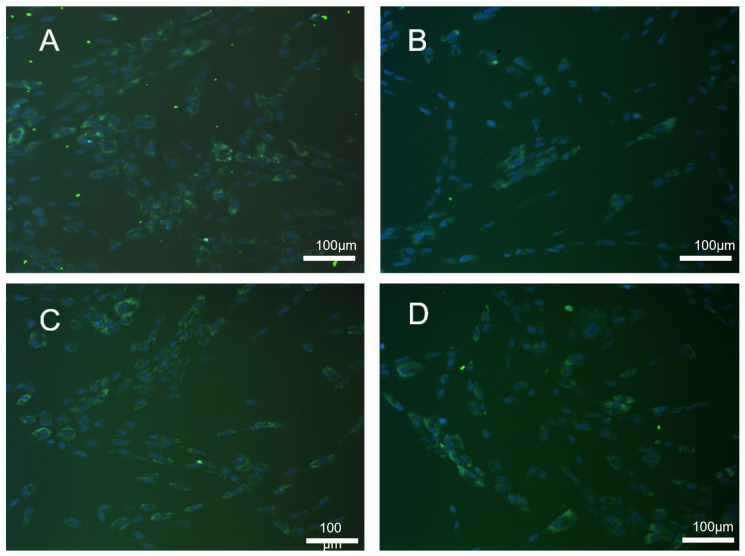
Immunofluorescence: SHED uptake of PLGA and PLGA/chitosan nanoparticles following 24 h exposure (blue DAPI staining was used for nuclei, while NPs are labeled with FITC and appear in green). (**A**) Normal SHED PLGA uptake; (**B**) CD117+ SHED PLGA uptake; (**C**) normal SHED PLGA/chitosan uptake; (**D**) CD117+ SHED PLGA/chitosan uptake. Magnification ×20; nanoparticle concentration 5 µg/mL.

**Figure 5:**
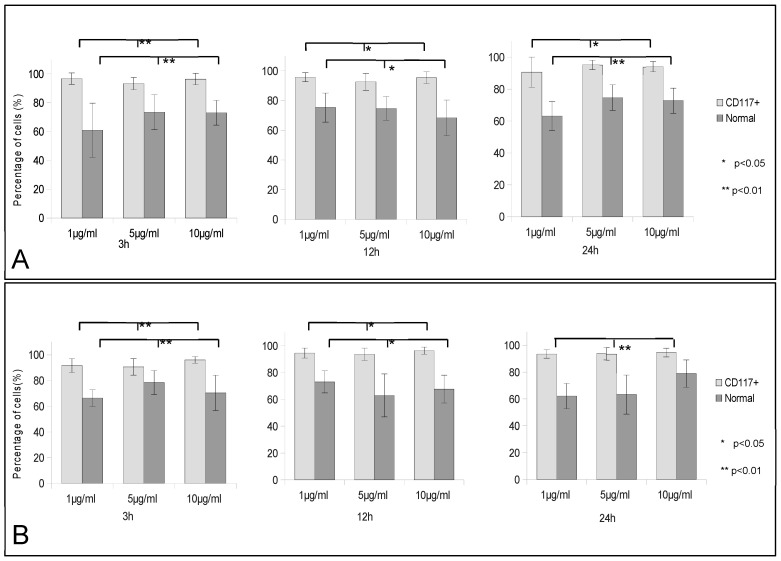
Uptake of polymeric nanoparticles by normal and CD117+ SHEDs at various NP concentrations and different time points. (**A**) PLGA uptake by normal and CD117+ SHEDs; (**B**) PLGA/chitosan uptake by normal and CD117+ SHEDs. Statistical significance was set at * *p <* 0.05 and ** *p <* 0.01 ANOVA; n = 5 independent experiments.

The uptake of nanoparticles was monitored by immunofluorescence (Figure 4). Analysis of the SHED cells uptake of PLGA (Figure 5A uptake graph) showed around 60% to 73% of nonseparated cells uptake of nanoparticles after 3 h of incubation. Over time, the percentage of fluorescent cells increased to about 75% of the cells, and, until the end of the incubation at 24 h, remained stable with one exception—the concentration of 1 µg/mL dropped back to 64%. Most stable was the uptake of 5 µg/mL PLGA NPs after 3 h of incubation at 73.37% ± 12.11%, 12 h of incubation at 74.67% ± 7.97%, and 24 of incubation at 74.70% ± 5.00%.

A statistically significant difference was found in the CD117^+^ fraction uptake compared with the same conditions for the nonseparated SHEDs. The percentage of cells uptake for PLGA NPs remained stable around 95% throughout the entire incubation for all concentrations. Nonseparated SHEDs expressed similar uptake rates for the PLGA/chitosan NPs (Figure 5B uptake graph). Between 66% and 75% of the cells showed green fluorescence. The uptake of 1 and 5 µg/mL PLGA/chitosan NPs dropped at 24 h compared with 3 h of incubation, respectively, to 62.15% ± 9.43% from 66.35% ± 6.41% and to 63.24% ± 14.6% from 75.45% ± 9.43%, as the uptake of 10 µg/mL increased from 70.40% ± 13.76% to 75.93% ± 10.17%. The CD117^+^ fraction showed 91.76% ± 5.35% at 1 µg/mL; 90.67% ± 6.39% at 5 µg/mL and 96.10% ± 2.55% at 10 µg/mL after 3 h of incubation with PLGA/chitosan NPs. At the end of the incubation, the number of fluorescent cells increased to 93.55% ± 3.10% at 1 µg/mL and 93.75% ± 4.67% at 5 µg/mL, and dropped to 94.65% ± 3.26% at 10 µg/mL. Significantly more cells were showing NPs uptake from CD117^+^ compared with the nonseparated SHEDs.

## 4. Discussion

One major challenge when addressing stem cell therapy is finding adequate scaffolds or careers knowing that tissue regeneration requires an optimal combination of cells and active molecule growth factors. Therefore, a wide range of organic and inorganic materials are currently applied in various in vitro, preclinical, and clinical studies. A novel method for active substances in tissue regeneration is the use of nanoparticles. Among these, polymeric NPs have recently been introduced as drug delivery vehicles, as their application has resulted in improved pharmaceutical bioavailability, enhanced efficacy, and reduced toxicity [13]. Poly (d,l-lactide-coglycolide acid) (PLGA) is a widely investigated polymer for NP synthesis [14]. It is able to improve the oral bioavailability of DNA and peptides [15]. Chitosan is another biocompatible nontoxic polymer. It is an N-deacetylated derivate of chitin known for its ability to promote sustained release of various proteins, hydrophilic and hydrophobic drugs, and DNA transfection [16]. Thus, PLGA nanoparticles are broadly accepted drug carriers due to their biocompatibility and ability to provide sustained, controlled, and targeted drug delivery.

The body recognizes particles as foreign bodies, and the reticulo-endothelial system (RES) eliminates them from the blood. Additional surface modification, i.e., PEGylation, can increase PLGA nanoparticles’ half-life and prolong their circulation in the blood stream.

Passive transport and active endocytosis are well-known methods for cellular uptake of nanoparticles. Two main types of endocytosis have been described in the literature: nonspecific endocytosis and receptor-mediated endocytosis, which includes clathrinid-mediated endocytosis and caveolae-mediated endocytosis [17]. The pathway of cellular uptake depends on the surface characteristics and size of the nanoparticles. Preparation conditions are of high importance for the biological applications of nanoparticles. Surface charges of nanoparticles affect their cellular internalization. Positively charged nanoparticles are expected to have more sustained cellular uptake due to the positively charged surface of the nanoparticle and negatively charged cell membrane. PLGA nanoparticles are known to have negative charges as well as the cell surface. The particles’ negative charge can be shifted to neutral, or even positive, by surface modification, including PEGylation (polyethylene glycol (PEG) or chitosan coating [18]. It is well-known that chitosan also possesses absorption-enhancing properties and is able to accelerate cell uptake.

Within the past few years, mesenchymal stem cells have been widely discussed as a potential cancer gene therapy vehicle [19]. The combination of stem cells and loaded particles may lead to even better results in anticancer therapy. In the present study, the cellular uptake of fluorescent nanoparticles by OKSCs and SHEDs was identified by semiquantitative immunofluorescence. PLGA and PLGA/chitosan nanoparticles were not loaded with active molecules and drugs. The nanoparticles in the current experiment were spherical in shape with a smooth surface. The aim was just to assess the differences in cellular uptake after stem cells’ incubation with various concentrations and durations of incubation time. We included two different fractions of SHED cells in the experiment: nonseparated SHEDs and a CD117+ fraction. CD117 is a well-known stem cell marker, previously applied in in vitro studies for magnetic and fluorescent activated cell sorting. The marker is expressed in wide range stem cell cultures. Our results revealed that 5 and 10 µg/mL PLGA uptake remained stable in both cell fractions at the 3rd, 12th, and 24th hour of incubation. When these concentrations are applied, the uptake is not time dependent. For the nonseparated SHEDs incubated with 1 µg/mL PLGA, the uptake was significantly increased at the 12th hour of incubation, whereas for the CD117+ fraction in the same conditions, the uptake decreased. The same tendency was observed in cells incubated with 1 µg/mL PLGA/chitosan. In the normal nonseparated SHED fraction, the uptake significantly decreased at the 12th hour of incubation with 5 and 10 µg/mL PLGA/chitosan. The optimal PLGA and PLGA/chitosan nanoparticles’ uptake for both SHED cell fractions occurred at a concentration of 1 µg/mL; the optimal PLGA and PLGA/chitosan incubation time for nonseparated cells was 3 h, and for the CD117+ cells, it was 12 h. Therefore, the cell uptake mechanism depends on the cell fraction, incubation time, and surface properties of the nanoparticles.

The present study established, for the first time, a baseline for PLGA-NP uptake by two subpopulations of oral stem cells, oral keratinocyte stem cells, and dental stem cells. We report important uptake parameters such as particle size, particle chemical composition, time, and concentration tailored for each studied cell population. However, more studies are needed to certify these parameters, not only in an in vitro setting, but also in vivo.

## Figures and Tables

**Table 1:** PLGA and PLGA/Chi polymeric nanoparticles’ characteristics.

	Size nm	PI	Zeta Potential mV	PVA %
PLGA	95 ± 2	0.11 ± 0.019	−38 ± 2	25.8 ± 0.5
PLGA/Chi	132 ± 4	0.38 ± 0.027	+76 ± 2	24.1 ± 0.8

Note: sample pH 5.5.

## Data Availability

Not applicable.
